# What are interesterified fats and should we be worried about them in our diet?

**DOI:** 10.1111/nbu.12264

**Published:** 2017-05-08

**Authors:** C. E. Mills, W. L. Hall, S. E. E. Berry

**Affiliations:** ^1^ King's College LondonLondonUK

**Keywords:** interesterified fats, fat spreads, health

## Abstract

Interesterified (IE) fats are used in a wide range of food products and were introduced as a replacement for *trans* fats, which are known to be detrimental to cardiovascular health. However, the effects of interesterification on metabolism and subsequent effects on cardiovascular health are not understood and previous studies have seldom investigated industrially‐relevant IE fats. No legislation currently exists regarding the labelling of IE fats in food products and therefore estimates of average consumption rates in the UK population are currently unavailable. In order to meet the urgent need for a systematic investigation of the health effects of consumer‐relevant IE fats, it is essential to estimate current IE fat intakes and to investigate biological mechanisms that might mediate acute and chronic cardiometabolic effects of commercially relevant IE fats.

## Introduction

*This article reviews the background and current knowledge forming the rationale for the new Biotechnology and Biological Sciences Research Council Diet and Health Research Industry Club (BBSRC‐DRINC)‐funded project on the health impact of interesterified fat*.

Cardiovascular disease (CVD) remains one of the biggest public health concerns globally and there is substantial evidence that following dietary guidelines on dietary fatty acid intakes may reduce risk of CVD events (Oh *et al*. 2005; Toledo *et al*. 2013; Reidlinger *et al*. 2015). Much research and discussion have been devoted to the strength of the evidence for the health benefits of replacing saturated fatty acids with unsaturated fatty acids and the role of reducing dietary *trans* fatty acids in lowering CVD risk, which is well established (Mozaffarian *et al*. 2006). Artificial *trans* fats (generated from vegetable oils that have undergone partial hydrogenation to turn them into solid or semi‐solid fats) were originally introduced as a cheaper and healthier alternative to animal fats. However, levels of artificial *trans* fat in the UK diet have reduced markedly as UK food manufacturers have moved away from using these as a source of hard vegetable fat in their products (Stender *et al*. 2014). Interesterified (IE) fats are now used as a hard fat replacement for *trans* fats in a large range of commonly consumed foods, including spreads, bakery and confectionary products. Similar to artificially produced *trans* fats, there is no legal requirement for food manufacturers to include IE fats on food labels. Thus, without collaboration from the food industry, it is hard to approximate how much is being consumed. Estimates from US data suggest that approximately 3% of energy intake would come from IE fats if they were the sole replacement hard fat for partially hydrogenated vegetable oil (Lefevre *et al*. 2012). The drive for a reduction in industrial *trans* fats in food products was prompted by evidence indicating associations between consumption and undesirable cardiovascular health effects (Mozaffarian *et al*. 2006). IE fats were introduced as a suitable replacement as they can provide desirable functional properties, related to the product's melting point, texture and shelf stability. Without such fats, these products would need to be made from high‐saturated fat products, such as butter or lard, otherwise they would have very different textural characteristics; consider the textural and stability challenges in manufacturing biscuits made from rapeseed oil – a softer, less shelf‐stable product would result.

Despite IE fat being widely used in food products in the UK and worldwide for decades, the impact on cardiovascular health is unknown. The use of IE fats instead of animal fats is assumed to be beneficial as it should help meet the population target for saturated fatty acids intake of <11% daily energy intake (Department of Health 1991), but there has been no systematic attempt to establish whether this is the case. With the relatively recent reformulation of products containing *trans* fat, it is important for industry to be confident in the fat replacements that they are using and, therefore, research in this area is a matter of priority. Available research to date has not assessed the health effects of the type of IE fats that are used in UK food products. Herein, we will discuss current understanding of IE fat metabolism and its impact on cardiovascular health. We will also present our new research project, which will engage major food industry stakeholders in knowledge exchange partnerships to identify the most industrially relevant IE fats. Finally, we will consider future priority areas of research in this field.

## What are interesterified fats and why does the food industry use them?

**Figure 1:**
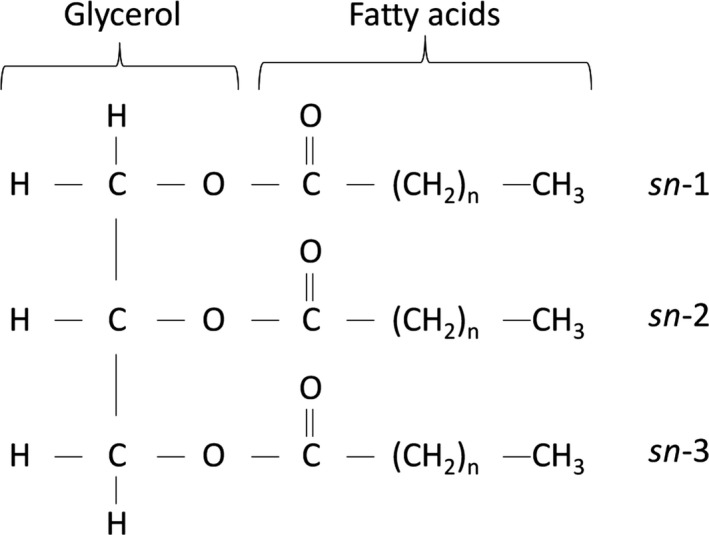
Triacylglycerol (TAG) structure showing glycerol with three fatty acids.

*What?* Dietary fats consist predominantly of triacylglycerols (TAG) (~95% of dietary fat), which are formed of three fatty acids esterified to a glycerol backbone. Fatty acids are positioned in one of three places on the glycerol molecule and labelled using a stereospecific numbering system (*sn*); *sn*‐1 and *sn*‐3 refer to the fatty acids on the outer positions and *sn*‐2 is the fatty acid in the middle (Gurr *et al*. 2016) (Fig. 1).

**Figure 2:**
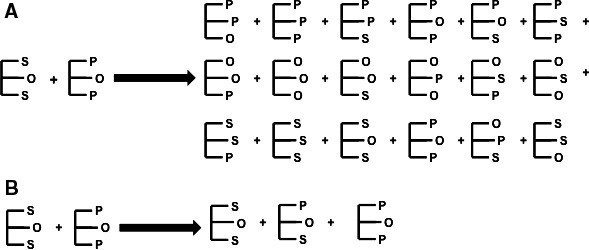
Simplified schematic representing random (A) and directed (B) interesterification of palmitoyl‐oleoyl‐palmitoylglycerol (palmitic, oleic, palmitic acids; POP) and stearoyl‐oleoyl‐stearoylglycerol (stearic, oleic, stearic acids; SOS)

The nature of the fatty acids within the TAG determines the physical and (bio)chemical properties of the TAG; for example, the length of the fatty acid chains, level of unsaturation and, if unsaturated, the position (*e.g. n*‐3, *n*‐6, *n*‐9), and geometry of the double bonds (*cis* or *trans*). Interesterification is the process of swapping or rearranging fatty acids within a TAG (between the *sn*‐1, 2 and 3 positions) or between TAGs, by either chemical (giving random esterification, where fatty acids are moved to unspecified positions) or enzymic (which can give random or directed esterification, where fatty acids can be moved to specific positions) means. Figure 2 shows a simplified schematic of random (A) and direct interesterification (B). This reshuffling of fatty acids produces different TAG molecular species and can be used to create fats with either a specific positional composition (*e.g*. Betapol^®^, an infant formula product, which mimics the positional composition of human breastmilk) or physical properties (*e.g*. modifying the melting point and solid fat content, without increasing the levels of saturated fatty acids). The commercial interesterification process used by the food industry for spreads, bakery and confectionary products reduces the saturated fatty acid content of the fat by approximately 10% compared to a non‐IE fat with a similar solid content. In Europe, palmitic acid‐based fats are commonly interesterified using combinations of palm oil fractions (palm kernel and palm stearin) to generate an IE hard stock. This IE hard stock is then blended at different ratios (depending on the final application and required solid fat content) with a vegetable oil, such as rapeseed or linseed oil. Plant and animal fats have different structural compositions as can be seen in Table 1 (Small 1991; Berry 2009). Plant fats typically have higher proportions of saturated fatty acids at *sn‐*1 and *sn‐*3, whereas animal fats have higher proportions of saturated fatty acids at *sn*‐2. Hence, industrial interesterification of plant fats will often yield TAGs with increased proportions of saturated fatty acids at the *sn*‐2 position as well as modifying the melting properties of the fat.

**Table 1:** Details of abundant triacylglycerol molecular species in various foods

	% total fat saturated	Major triacylglycerols a
Breastmilk	44	OPO, OPP
Betapol^®^ (formula milk)	44	OPO
Cow's milk	50	POO, PPO, OPO
Butter	63	PPB, PPC, POP
Lard	44	SPO, OPL, OPO
Palm oil	49	POP, POO, POL
Rapeseed oil	7	OOO, LOO, OOLn
Linseed oil	9	LnLnLn, LnLnL, LnLnO

B is butyric acid, 4:0; C is capric acid, 10:0; L is linoleic acid, 18:2, Ln is linolenic acid, 18:3; O is oleic acid, 18:1; P is palmitic acid, 16:0; S is stearic acid, 18:0 [Sources: Berry (2009); Small (1991)]

a Written as *sn‐1 sn‐2 sn‐3*

*Why?* The UK government has long recommended that saturated fatty acids in the diet should be reduced (Department of Health 1991). In a bid to reduce high‐saturated fat foods, the food industry has historically utilised structural geometry changes in partially hydrogenated vegetable oil to create *trans* fatty acids, which increases the melting point of the vegetable oil. Partially hydrogenated vegetable oils are shelf‐stable, have a high solid fat content and replicate the physical properties of high melting point saturated fats. However, high *trans* fatty acid content has since been shown to be detrimental to cardiovascular health; for example, a recent meta‐analysis showed associations between consumption of *trans* fat and both total and coronary heart disease mortality (relative risk 1.21 and 1.28, respectively). This was found to be specifically related to industrially produced (or artificial) *trans* fatty acids, rather than the natural (*i.e*. ruminant) *trans* fatty acids (de Souza *et al*. 2015).

Blending fats, fractionation, full hydrogenation (which does not yield *trans* fatty acids), interesterification or genetic modification are all viable alternatives to *trans* fat. However, none of these processes alone produce fats with suitable functionality for most of the food applications required and the combined process of interesterification of blends of fractionated fats or fully hydrogenated fats appears to be the best option available for the food industry. In the UK, there is currently a legal requirement for clear labelling of hydrogenated fats in food products. However, labelling does not distinguish between fully or partially hydrogenated fats. Therefore, consumers do not differentiate between fully hydrogenated and partially hydrogenated (*trans*) fat and simply deem hydrogenated fats as ‘bad fats’. Consequently, the UK food industry has been driven away from using full hydrogenation as consumers avoid it. Therefore, IE fractionated fats are the most commonly used method by the UK food industry.

## Estimating intake of interesterified fat

It is difficult to accurately estimate intakes of IE fat in the UK population as there is no requirement for IE fats to be labelled on food products and analysis of the IE content in foods is a complex, technically challenging process. To date, no estimations of dietary IE fat intake in the UK have been made. Lefevre *et al*. (2012) modelled data from the *National Health and Nutrition Examination Survey* (*NHANES*) to predict intake of IE fully hydrogenated soya bean oil (a stearic acid‐rich IE fat) in the US if used as a (application appropriate) replacement hard fat for *trans* fats. They estimated that IE fat intakes in the *NHANES* population would be approximately 3% of daily energy (Lefevre *et al*. 2012). They also predicted that replacing *trans* fats with a fully hydrogenated stearic acid‐rich IE fat would reduce CVD risk by 1.2%. However, since these results were based on stearic acid‐rich IE fat, they cannot be extrapolated to the UK population, as in the UK *trans* fats have already been replaced with palmitic acid‐rich IE fats (Lefevre *et al*. 2012).

In the light of this, there is a pressing need to estimate the amount of IE fat consumed in the UK, based on the main IE fat blends used by the food industry, in order to assess the potential public health effects of their use in place of other application appropriate fats. To estimate UK intakes of IE fat, a similar approach to that taken by Lefevre *et al*. (2012) would need to be applied to the *National Diet and Nutrition Survey* (*NDNS*) population data. Such estimates would inform the design of clinical trials to test the health effects of IE fat; this is an area that our group are currently working on as part of a recently awarded BBSRC‐DRINC grant, as discussed below.

## Does interesterification of palmitic acid‐rich fats influence their digestion, lipid metabolism or impact on cardiovascular health?

The impact of fat consumption on health, particularly cardiovascular health, is a topic that is regularly under debate. Although there is general consensus regarding the effect of degree of saturation and molecular geometry of fats on cardiovascular health, the influence of the specific arrangement of the fatty acids on the glycerol backbone on lipid metabolism and cardiovascular physiology is not understood. As with molecular geometry, there is a possibility that positional composition may affect dietary fat metabolism, digestibility and subsequent effects on cardiovascular health [as discussed in depth previously by our group (Berry 2009)]; however, the data are not consistent nor complete, with most studies using fats which are not commercially relevant.

Historically, it was believed that animal fats were more atherogenic than plant fats due to their positional composition (*e.g*. a higher proportion of saturated fatty acids in the *sn*‐2 position), despite, in some incidences, having a similar saturated fatty acid content (*e.g*. palm oil and lard; see Table 1). A plausible explanation for this is differences in digestion due to the specificity of pancreatic and lipoprotein lipase. These enzymes preferentially hydrolyse fatty acids at the *sn‐*1 and *sn‐*3 positions of TAG, which are released as free fatty acids, leaving a 2‐monoacylglycerol (2‐MAG: glycerol backbone with saturated fatty acid at *sn*‐2) (Nilsson‐Ehle *et al*. 1973; Yang & Kuksis 1991). Animal and human infant studies show that high melting point saturated fatty acids (such as stearic and palmitic acids) are poorly absorbed as free fatty acids compared to when they are present as 2‐MAG. Therefore, it has been suggested that industrial interesterification of plant fats (which increases the proportion of saturated fatty acids in the *sn*‐2 position, see Fig. 2) may improve the absorption/digestibility of saturated fatty acids. Indeed, this has been demonstrated in animals (Tomarelli *et al*. 1968; Mattson *et al*. 1979) and human infants (Filer *et al*. 1969; Carnielli *et al*. 1995), which suggests that saturated fatty acids consumed in products containing industrially IE fats are better digested, absorbed and cleared more quickly from circulation than those which contain saturated fatty acids at *sn*‐1 or *sn*‐3. This could have additional implications for health as these saturated fatty acids containing 2‐MAG may cause downstream effects due to differential incorporation into phospholipids, or differential delivery to the liver and peripheral tissues. However, animals lack, and human infants have low levels of, pancreatic and lipoprotein lipases, which will have an impact on the digestion and subsequent absorption and metabolism of TAG. Indeed, human adult studies demonstrate that saturated fatty acids are well absorbed regardless of their position (Shahkhalili *et al*. 2000) and that position does not differentially influence rates of metabolism (Summers *et al*. 1998, 1999). There are insufficient data to draw sound conclusions regarding the effect of IE fat on metabolism and digestibility in humans, specifically those used commercially in the UK and Europe.

**Figure 3:**
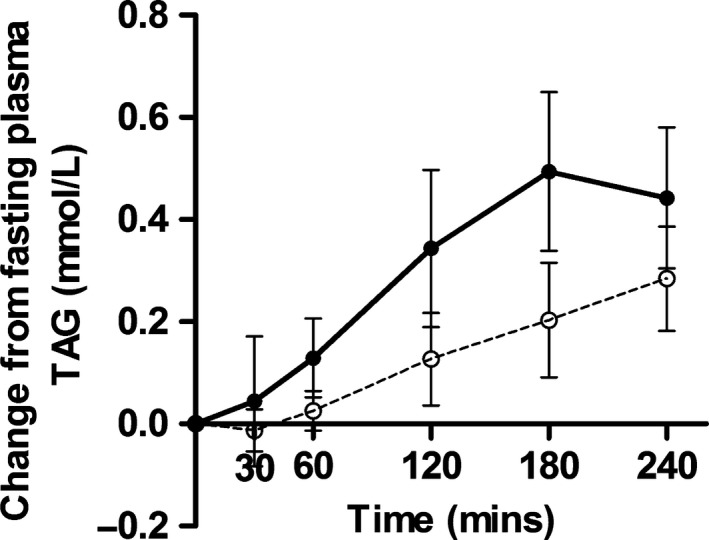
Mean change in plasma triacylglycerol (TAG) and 95% CI after test meals containing non‐interesterified (*open circles*) or interesterified (*filled circles*) fats; *P* < 0.009 [Source: Hall *et al*. (2016)].

Although it is important to consider the long‐term health effects of dietary fats, assessing acute responses provides insight into whether there is likely to be an impact on lipoprotein metabolism which underpins many of the chronic cardiometabolic effects of dietary fat (Jackson *et al*. 2012). The duration and peak of postprandial lipaemia, a physiological response to fat‐containing meals that is characterised by a rise in blood TAG concentrations 2–8 hours after a meal, are considered independent markers of CVD risk. Similar to the research on metabolism and digestibility, there is little consensus on the impact of IE fats on postprandial lipaemia. Most studies have observed a reduction (Yli‐Jokipii *et al*. 2001; Berry & Sanders 2003; Sanders *et al*. 2003) or no change (Zampelas *et al*. 1994; Summers *et al*. 1998, 1999; Yli‐Jokipii *et al*. 2003; Berry *et al*. 2007a,b) in postprandial lipaemia in response to IE fats. However, these studies used IE fats that are rarely used by the UK food industry. Using the most commonly consumed IE palmitic acid‐rich fat, our group recently reported that an IE palm kernel/palm stearin hard stock (80:20 blend) increased postprandial lipaemia compared to the non‐IE equivalent fat; see Figure 3 (Hall *et al*. 2016); this is contrary to all previous studies using commercially irrelevant IE fats. However, this pilot study assessed postprandial effects for only 4 hours and longer term postprandial effects need to be characterised, as both the duration and the peak lipaemic response are important in influencing CVD risk factors.

## Ongoing research and future directions

Commercial IE fats may make a significant contribution to the UK diet, but evidence to date on the cardiovascular health effects of IE fats is largely based on studies using IE fats that are not used commercially. Therefore, there is an urgent need to systematically investigate the estimated intakes and health effects of food industry‐relevant IE fats. Areas of uncertainty that should be addressed include mechanisms determining the postprandial handling of IE fats, acute and chronic cardiometabolic effects of commercially relevant IE fats at relevant doses and the differences between the cardiovascular health effects of stearic acid‐ and palmitic acid‐rich IE fats.

Preliminary research from our group found that one of the most commonly consumed IE fat blends when incorporated into high‐fat test meals led to increased blood TAG concentrations up to 4 hours post‐consumption, compared to the equivalent non‐IE fat (Hall *et al*. 2016). Large rises in blood lipids after meals are an important risk factor for CVD. Our new BBSRC‐DRINC grant ‘The health impact of industrial interesterification of dietary fat’ will investigate the cardiometabolic health effects of the most widely consumed IE fats to explore our initial findings in more detail. The project will be run at the Division of Diabetes and Nutritional Sciences at King's College London and is organised into three work packages. Work package 1 will estimate the dietary intake of IE fat in the UK using an existing database of dietary intakes (*NDNS,* years 2013‐2014), which encompasses a large, nationally representative study population, in combination with information on IE fat contents of foods provided by industry. This information will be used to predict the likely impact on blood lipid profiles if current dietary intakes of IE fats were replaced by traditional hard fats, such as butter and lard. This will provide the first ever data on the potential public health impact of including these fats in the UK food supply. Work package 2 will involve a detailed human study to assess the digestion, absorption and metabolism of the most commonly consumed IE fat, compared to the non‐IE equivalent fat. Work package 3 will include a second human study to determine whether blends of different proportions of IE fats (*i.e*. the proportions that are most commonly consumed in products such as ‘healthier’ spreads and bakery fats) have any effects on markers of cardiometabolic risk [such as postprandial lipaemia, flow mediated dilatation (FMD), nitric oxide bioavailability], compared to control oils/fats containing 0 and 100% IE fat.

This work will provide a better understanding of the potential cardiovascular health impact of current intakes of IE fat and will form the basis of much needed chronic dietary intervention trials designed to explore the effects of IE fats on public health.

Scientific evidence on the potential health effects of consuming IE fats will be particularly relevant to the food industry, nutritionists, dietitians, other health professionals and government policymakers and may eventually be translated into national dietary guidelines.

## Conflict of interest

SEEB has previously received research funding from Malaysian Palm Oil Board. WLH and CEM have no conflicts of interest to declare.
